# Neoadjuvant Treatment in Locally Advanced Thyroid Carcinoma

**DOI:** 10.3390/jcm13195769

**Published:** 2024-09-27

**Authors:** Víctor Navas Moreno, Fernando Sebastián Valles, Marcos Lahera Vargas, Berta Hernández Marín, Elena Carrillo López, Mónica Marazuela, José Luis Muñoz de Nova

**Affiliations:** 1 Department of Endocrinology and Nutrition, Hospital Universitario de La Princesa, 28028 Madrid, Spain; 2 Instituto de Investigación Sanitaria Princesa (IIS-IP), 28028 Madrid, Spain; 3 Department of Oncology, Hospital Universitario de La Princesa, 28028 Madrid, Spain; 4 Department of General and Digestive Surgery, Hospital Universitario de La Princesa, 28028 Madrid, Spain; 5 Department of Surgery, Universidad Autónoma de Madrid, 28029 Madrid, Spain

**Keywords:** thyroid cancer, neoadjuvant treatment, targeted therapies, advanced thyroid cancer, thyroidectomy

## Abstract

Although the focus in the last decades has been on the overdiagnosis of incidentally detected thyroid carcinomas in early stages, the other extreme of the disease is represented by locally advanced tumors with the invasion of neighboring structures. These are infrequent tumors, but they have a high complexity and a poor prognosis. In the absence of effective therapies allowing preoperative tumor reduction, in order to achieve a more restricted surgery, treatment was limited to aggressive surgery with resection of the aerodigestive tract and major vascular structures or palliative treatment. However, due to the increased knowledge of tumor biology and the results that tyrosine kinase inhibitors have achieved in the treatment of radioactive iodine-refractory tumors, neoadjuvant therapy with a curative intent has emerged as a reality to be taken into account when dealing with these patients. This paper presents a narrative review of the current scientific evidence regarding neoadjuvant treatment in locally advanced thyroid cancer.

## 1. Introduction

In the past few decades, there has been a dramatic increase in the diagnosis of patients with thyroid cancer, but this has not translated into an increase in the mortality associated with this tumor, which remains relatively low [1]. Although its incidence is quite low (<5%), it is estimated that 50% of deaths due to thyroid carcinoma are caused by locally advanced tumors with or without associated distant metastases [2,3].

In the absence of effective therapy to address these cases, surgery remains the treatment of choice in most of them. The problem arises when tumor invasion occurs over unresectable organs (spine or mediastinal great vessels) or organs that are resectable but subject to complex surgery and susceptible to serious complications (carotid artery or visceral axis) [3,4,5,6]. Moreover, even after successful surgery, the patient usually faces significant sequelae, for example, when a laryngeal resection is performed or even when a laryngeal recurrent nerve is sacrificed, with a decrease in quality of life.

If we consider the different subtypes of thyroid cancer independently, the frequency of locally advanced tumors varies substantially. In differentiated thyroid cancer (DTC), this is an infrequent situation (<2%), except in widely invasive follicular carcinomas, which may be due to both the primary tumor and the extranodal component of nodal metastases [3]. In the context of medullary thyroid carcinoma (MTC), given its high propensity to develop lymph node metastases, some of which are mediastinal in location, it is not uncommon for vascular invasion to derive from them [3]. Finally, anaplastic thyroid carcinoma (ATC) is a very aggressive entity, in which the invasion of locoregional structures is very frequent at diagnosis, being the main cause of death in these patients [7].

However, in recent years, a battery of treatments based on tyrosine kinase inhibitors (TKIs), such as sorafenib or lenvatinib, have emerged and have achieved excellent results in the field of iodine-refractory tumors. These results have led to attempts to apply this success to the field of neoadjuvant therapy, with the aim of achieving a tumor reduction that at least allows less mutilating surgery and a better prognosis for the patients [8].

The scientific evidence in neoadjuvant treatment of thyroid cancer is still limited, and it is unlikely that the low frequency of these tumors will allow us to perform prospective randomized trials. This article reviews the available evidence in the three main clinical scenarios: differentiated thyroid carcinoma, medullary thyroid carcinoma, and anaplastic thyroid carcinoma.

## 2. Differentiated Thyroid Carcinoma

DTC encompasses carcinomas originating from follicular cells, including papillary thyroid carcinoma and follicular thyroid carcinoma [9]. These neoplasms constitute the vast majority of thyroid cancers (>90% of all thyroid malignancies) [10,11]. In recent decades, an increase in the incidence of DTC has been observed, largely attributable to the more frequent use of cervical imaging and ultrasound-guided fine-needle aspiration (FNA) cytology. Notably, this increase in incidence has not been accompanied by a rise in mortality, suggesting the potential for the overdiagnosis and overtreatment of DTC with a favorable prognosis [1]. Consequently, there is a growing trend toward more conservative management approaches [10,12]. However, a concurrent rise in the incidence of advanced DTC has also been reported, indicating that the increase in diagnostic testing does not fully account for the observed rise in DTC incidence [13,14].

Locally advanced DTC refers to tumors that invade extrathyroidal structures, potentially causing compressive symptoms [15]. This condition is estimated to occur in up to 15% of DTC cases, with a higher prevalence in older patients and in those with larger tumors [15]. Its significance lies in the fact that the prognosis differs markedly from that of non-invasive DTC [15,16,17]. Historically, the management of advanced DTC in resectable cases involved extensive surgery followed by adjuvant therapy, which often resulted in considerable postoperative morbidity [18,19]. In cases that were unresectable or involved distant metastases, the treatment options were extremely limited, as both chemotherapy and radiotherapy have limited evidence supporting their efficacy and are typically palliative, aimed at decreasing tumor progression [15,18].

Due to its low prevalence, the ability to conduct prospective studies on various therapeutic strategies for advanced DTC is significantly limited. However, there is growing evidence from some phase II clinical trials and case series supporting the use of TKIs in neoadjuvant therapy in advanced DTC cases. This approach has enabled the complete surgical resection of tumors that were initially considered unresectable and has also been shown to reduce morbidity and mortality by avoiding more aggressive surgical interventions.

### 2.1. Radioactive Iodine Therapy

Although radioiodine ablation is one of the most widely used treatments in the management of thyroid cancer, its use for neoadjuvant purposes is clearly limited by the presence of thyroid tissue. Only one case has been reported involving a 57-year-old woman with unresectable 6 cm papillary thyroid carcinoma presenting with compressive symptoms. She received three doses of radioactive iodine (total cumulative dose of 270 mCi), resulting in a reduction of the tumor diameter to 3 cm. Total thyroidectomy was performed 20 months after the initiation of RAI therapy [20]. No other experiences have been reported to date. In summary, radiation therapy is not considered as a neoadjuvant option due to its potential to cause cervical fibrosis, which can complicate subsequent surgical procedures [21].

### 2.2. Chemotherapy

The use of conventional chemotherapy regimens as neoadjuvant therapy is not recommended in the guidelines for DTC [10].

In metastatic DTC, doxorubicin was initially approved based on preliminary evidence of a partial response. However, the subsequent evidence, both for monotherapy and in combination with cisplatin, has been controversial, and there is currently no solid evidence that the response is significant and cost effective [21,22,23,24].

In response to this conflicting evidence in metastatic DTC, the use of neoadjuvant chemotherapy in advanced DTC cases was explored. A Phase 2 trial was published in 2012 involving 29 patients with follicular thyroid carcinoma or Hürthle cell carcinoma [25]. Their results suggested that treatment with vinblastine/vincristine combined with doxorubicin or other regimens might be effective. Their study reported a response rate (reduction in tumor size of >50%) of 44.8%, with surgical outcomes showing R0 (no residual tumor) in 51.7% of cases, R1 (microscopic residual tumor) in 34.5%, and R2 (macroscopic residual tumor) in 13.8%. Using similar criteria and therapeutic regimens, the same team published data in 2013 for 16 patients with papillary thyroid carcinoma, reporting a response rate of 40.0%. The surgical resection outcomes were R0 in 12.5% of cases, R1 in 62.5%, and R2 in 25% [26]. Additionally, a series of two cases [27] were reported of squamous cell papillary thyroid carcinoma treated with paclitaxel. One patient, with distant disease, had a partial local response but eventually succumbed to metastases despite subsequent surgery and chemotherapy; the other patient, with only local disease, underwent surgery and remained alive for more than 14 and 22 months after the diagnosis, respectively.

### 2.3. Targeted Therapies

In recent years, TKIs have emerged as prominent therapeutic agents. These drugs target the molecular pathways involved in DTC pathogenesis, including RET, BRAF, vascular endothelial growth factor receptors (VEGFRs), fibroblast growth factor receptors (FGFRs), and platelet-derived growth factor receptors (PDGFRs) [28,29]. The use of TKIs has marked a significant shift in the management of RAI-refractory DTC, particularly with agents such as sorafenib, lenvatinib, and cabozantinib [30,31,32]. Their effectiveness has prompted investigations into their potential utility as neoadjuvant therapy in advanced DTC cases.

#### 2.3.1. Sorafenib

Sorafenib is an oral multikinase inhibitor drug targeting VEGFR-1, VEGFR-2, VEGFR-3, RET (including RET/PTC), RAF (including BRAFV600E), and PDGFR-β. Its pivotal clinical trial demonstrated efficacy in RAI-refractory DTC [33].

Regarding the use of sorafenib as neoadjuvant therapy, the first case [34] was reported in 2018 involving a 20-year-old male with papillary thyroid carcinoma presenting with a large cervical mass and suspected lymphadenopathy. Due to the initial unresectability, neoadjuvant treatment with sorafenib at 400 mg daily was initiated. After 13 months of treatment, there was a significant reduction in tumor size, which allowed for near-total thyroidectomy and cervical lymphadenectomy, followed by radiotherapy and RAI treatment. The patient remained asymptomatic with stable residual thyroid tissue at 52 months after diagnosis.

In 2019, another case [35] was reported involving a 32-year-old male with a 7.8 cm thyroid lesion invading the trachea and esophagus, with a surgical biopsy confirming papillary thyroid carcinoma. Sorafenib at 800 mg daily was administered for 6 months, resulting in a 70% reduction in tumor size and detachment from adjacent structures. This facilitated total thyroidectomy and adjuvant radioactive iodine treatment. At one-year follow-up, the patient showed incomplete biochemical response criteria but had negative imaging studies for recurrence.

#### 2.3.2. Lenvatinib

Lenvatinib is an oral multikinase inhibitor targeting VEGFR-1, VEGFR-2, VEGFR-3, FGFR 1-4, PDGFR-α, RET, and KIT signaling networks. In its Phase III trial in advanced disease, lenvatinib demonstrated efficacy in terms of progression-free survival and response rate compared to placebo [30].

In 2017, the first use of lenvatinib as a neoadjuvant therapy in advanced DTC [36] was reported. The patient was a 73-year-old male with an 18 mm thyroid mass and lymphadenopathy measuring 43 mm, invading the right internal jugular vein, and another lymph node of 30 mm invading the esophagus and trachea. Due to the complex surgical nature of the lymphadenopathy, lenvatinib at 14 mg daily was administered for 22 weeks, resulting in an 84.3% reduction in one lymph node and a 56% reduction in the other, enabling resection while preserving the esophagus. After 11 months, RAI treatment was initiated, and no distant metastases were observed at that time.

Another case [37] was reported in 2019, it was a papillary thyroid carcinoma invading the trachea, making it initially unresectable. The patient was treated with sorafenib at 800 mg daily but had to discontinue after one month due to adverse effects. Subsequently, lenvatinib was administered at 24 mg daily for 14 months, resulting in a reduction from 31 × 59 × 32 mm to 17 × 28 × 22 mm, which allowed for complete resection of the lesion and subsequent RAI treatment.

Another case [38] was published involving a 75-year-old woman with papillary thyroid carcinoma localized to the mediastinum with pulmonary metastases. The mediastinal mass measured 68 mm and obstructed the brachiocephalic trunk and the superior vena cava, posing risks for pulmonary embolism and superior vena cava syndrome. Treatment with lenvatinib at 14 mg daily for 16 weeks resulted in a reduction to 48 mm in the maximum axis, allowing for total thyroidectomy and resection of the mediastinal mass. Three months after surgery, the metastatic lesions had disappeared, and the mediastinal mass was completely resected.

Multiple equally successful clinical cases have been described, so that, although the clinical evidence is still very low, lenvatinib is currently the most established drug in the management of DTC with neoadjuvant intent.

#### 2.3.3. Apatinib

Apatinib is a selective oral inhibitor of VEGFR-2 that has demonstrated efficacy in solid tumors [39]. There is one case reported [40] involving a 64-year-old woman with a 7 cm papillary thyroid carcinoma compressing the trachea and esophagus, causing dysphagia. Treatment with apatinib at 500 mg daily was initiated. Within just 6 weeks, the tumor size decreased by more than 50%, and the dysphagia resolved. Subsequently, total thyroidectomy was performed with esophageal preservation, followed by RAI treatment. At 6 months, imaging studies showed no metastases, but a focal deposit in the neck was detected on ^131^I scanning.

#### 2.3.4. Anlotinib

Anlotinib is an oral multikinase inhibitor targeting VEGFR, FGFR, PDGFR, and c-Kit. Initially tested in MTC, it has also demonstrated efficacy in iodine-resistant DTC [41,42]. In 2021, a single-arm Phase II clinical trial [43] was published evaluating anlotinib as neoadjuvant therapy in patients with locally advanced and unresectable or difficult-to-resect thyroid cancer. R0/R1 resections were achieved in 8 of 13 cases.

#### 2.3.5. Surufatinib Combined with Toripalimab

Surufatinib is an oral multikinase inhibitor targeting VEGFR, FGFR-1, and colony stimulating factor-1 receptor (CSF-1R). Toripalimab is an immune checkpoint inhibitor targeting programmed cell death protein-1 (PD-1). A Phase II study [44] demonstrated the efficacy of these agents, allowing R0/R1 resections in 9 of the 10 patients included.

## 3. Anaplastic Thyroid Carcinoma

ATC is a rare malignant thyroid neoplasm, accounting for approximately 2% of thyroid cancers [45]. Although it originates from follicular thyroid cells, it is the most undifferentiated subtype of thyroid cancer and retains none of the biological characteristics of follicular cells. This type of cancer has an extremely poor prognosis, with a disease-specific mortality rate of nearly 100% [46], contributing to almost one-third of all thyroid cancer-related deaths [7]. The median overall survival in patients with ATC, with or without metastasis, is less than 6 months, and the 5-year overall survival rate is less than 10% [47]. Complete surgical resection, when feasible, has been shown to improve outcomes in ATC [48,49,50,51,52]. However, due to the rarity of the disease, it is not possible to conduct large-scale randomized studies in ATC, limiting the strength of recommendations in clinical practice guidelines regarding its pharmacological management.

Until a few years ago, there were little data suggesting that advanced neoadjuvant therapy could convert an initially unresectable primary tumor into a resectable one and optimize local tumor control. However, advances in molecular biology and the pharmacological inhibition of molecular pathways involved in tumor proliferation have increasingly provided evidence supporting the feasibility of a neoadjuvant approach in ATC. This approach offers the potential for surgical intervention in tumors that were previously deemed unresectable.

Recently, cancer immunotherapy has been developed with the aim of designing effective treatments to enhance the specificity and strength of the immune system against cancer [53]. The immune checkpoint proteins PD-1 and cytotoxic T-lymphocyte-associated protein 4 (CTLA-4) have been shown to act as brakes on the immune function, suggesting that immune checkpoint inhibition can reactivate T cells and more effectively eliminate cancer cells [54]. The pharmacological inhibition of molecular pathways involved in tumor proliferation has increasingly provided evidence supporting the viability of a neoadjuvant approach in ATC [55,56,57]. This approach offers the possibility of surgical intervention in tumors previously considered unresectable, as will be discussed further.

### 3.1. Chemotherapy

Some neoadjuvant chemotherapy regimens have been used in ATC. The ATA guidelines recommend adjuvant or neoadjuvant chemotherapy with combinations of (1) paclitaxel with carboplatin; (2) doxorubicin with cisplatin; (3) doxorubicin with docetaxel; or (4) paclitaxel alone or doxorubicin alone [58]. Unfortunately, chemoresistance is common in ATC, and survival outcomes are not very encouraging [59].

In a study involving 66 patients evaluating the response to paclitaxel [60], surgical resection was achieved in 43 patients, with an adequate response in 14. The median number of paclitaxel cycles in the surgically treated group was six. The median survival in the paclitaxel-treated group that achieved resection was 14.7 months. The response rate to paclitaxel was 23%, similar to that reported in clinical trials [58] and in prospective studies [59]. Patients who underwent resection after paclitaxel treatment had a significantly better prognosis, despite half of them having distant metastatic disease, suggesting that local control of ATC may improve survival. In this study, no associations were found between the paclitaxel response and the baseline characteristics, including inflammatory biomarkers. In another study with 138 patients [46], only 2 received paclitaxel with a neoadjuvant intent. In a case report [61], neoadjuvant doxorubicin in an inoperable ATC allowed sufficient tumor reduction to perform complete resection surgery, followed by adjuvant chemo- and radiotherapy, achieving a disease-free survival of at least 9.8 months.

### 3.2. Targeted Therapies and Immunotherapy

These tumors share a similar molecular pathogenesis to that observed in DTC, with alterations in genes such as BRAF [62], RAS, and NTRK, among others. The BRAF V600E mutation is present in 25–45% of ATCs [63,64]. As a permanently activated kinase, mutated BRAF V600E can phosphorylate its downstream targets, such as MEK and ERK, and is associated with aggressive features, such as lymph node involvement, large tumor masses, or extrathyroid metastases, leading to a poor prognosis [65]. Currently, the BRAF V600E status can be determined within days through immunohistochemistry or cfDNA sequencing [66].

The pharmacological inhibition of mutations involved in the pathogenesis of ATC is showing increasingly promising results. The combination of dabrafenib (a selective BRAF inhibitor) and trametinib (a selective MEK inhibitor) is now approved by the Food and Drug Administration (FDA) for BRAF V600E-mutant ATC [67]. Recently, some clinical cases [55,56,68] have been published proposing a short-term preoperative “neoadjuvant” therapy with BRAF-targeted therapies or, in BRAF-wildtype tumors, combinations of checkpoint inhibitors and TKIs in patients with unresectable ATC.

#### 3.2.1. BRAF-Mutated ATC

In 2018, the first case was reported [56] in which a neoadjuvant approach was applied to a patient with unresectable BRAF V600E-mutated ATC, achieving complete tumor resection after the administration of dabrafenib, trametinib, and pembrolizumab, with a survival of at least 16 months and a good quality of life. A case [55] of unresectable BRAF V600E-mutated ATC treated with pembrolizumab and lenvatinib for one month achieved complete surgical resection. A series of six cases [63] of BRAF V600E-mutated tumors treated with dabrafenib and trametinib with promising results achieved complete resection and locoregional control in 100% of cases. In a retrospective cohort study [68] that included 57 patients with BRAF V600E-mutated ATC treated with BRAF inhibitors (vemurafenib, dabrafenib, encorafenib) and MEK inhibitors (cobimetinib, trametinib, binimetinib), survival rates of 35.2 months were observed in the surgically treated group vs. 33.2 months in the neoadjuvant-treated group without surgery, with no statistically significant differences. However, the authors acknowledged a possible selection bias in the choice of therapeutic pathways in these patients.

#### 3.2.2. BRAF-Wildtype ATC

In BRAF-wildtype ATCs, multikinase inhibitors have also been tested [30]. Lenvatinib has been employed in neoadjuvant settings, achieving only a 2.2-month survival increase compared to palliative chemotherapy [69,70]. A case report of unresectable BRAF-wildtype ATC with programmed death-ligand 1 (PD-L1) expression >90%, in which treatment with lenvatinib and pembrolizumab for one month allowed tumor resection, achieved disease stability for at least 11 months [71]. Similarly, satisfactory responses were observed in a case of BRAF-wildtype ATC [72] treated off-label with lenvatinib and pembrolizumab, although another patient had to discontinue treatment due to pembrolizumab-induced bone marrow aplasia and passed away 5 months after diagnosis.

Immunotherapy with anti-PD-L1 antibodies, alone or in combination with BRAF inhibitors, has shown promising results for ATC treatment [73]. Several clinical studies have reported the use of camrelizumab (SHR-1210), a humanized, high-affinity, selective IgG4-κ anti-PD-1 monoclonal antibody, which has demonstrated good efficacy and acceptable safety in other solid tumors [57,74]. Yang et al. [65] reported a clinical case of a patient with BRAF-wildtype ATC treated with neoadjuvant famitinib and camrelizumab, achieving complete resection, locoregional control, and survival of at least 24 months from diagnosis.

Neoadjuvant treatment with BRAF/MEK inhibitors, immunotherapy, surgery, and adjuvant chemoradiation, followed by maintenance with BRAF/MEK inhibitors and immunotherapy, is currently being evaluated in clinical studies [57].

On the other hand, entrectinib was used as a neoadjuvant therapy in a case of unresectable ATC that presented an ETV6-NTRK3 fusion in its NGS [75]. Entrectinib was used prior to surgery, followed by radiotherapy, paclitaxel chemotherapy, and maintenance with entrectinib. In the reported clinical case, the patient started treatment with lenvatinib for 12 weeks, and upon tumor progression, entrectinib was initiated.

One such question is whether surgical intervention is necessary in patients whose disease is controlled with pharmacological therapy [68,72]. It is possible that, due to the short follow-up time in published cases, the phenomenon of resistance to pharmacological treatment is not fully observed. Nevertheless, considering that surgery after neoadjuvant therapy is safe and preserves quality of life, it remains a viable option [56,63,65,69,71,72,75]. The timing of preoperative treatment and subsequent maintenance is also a subject of debate. However, most studies recommend not discontinuing targeted therapies and immunotherapy after surgery, radiotherapy, and chemotherapy [56,63,65,71]. The coexistence of differentiated thyroid cancer alongside anaplastic cancer appears to be associated with a better prognosis, potentially allowing for the prediction of a favorable response [55,56]. This is a novel aspect that could help stratify risks and plan therapeutic pathways.

Lastly, further investigation into new therapeutic targets is necessary, such as EZH2 inhibition, which could be a promising neoadjuvant treatment for ATC. This approach has shown antitumor effects in vitro and in vivo and induces cellular differentiation [76], which could positively impact the survival and quality of life of patients with ATC.

## 4. Medullary Thyroid Carcinoma

MTC is a neoplasm that does not arise from follicular cells but from parafollicular or C cells derived from the neural crest. Therefore, although it has certain oncogenic pathways in common with follicular cells, it has unique features. The most important is the RET gene mutation, present in 100% of the hereditary and almost 90% of sporadic MTCs [77].

Although successful cases of surgical salvage after neoadjuvant treatment with multikinase inhibitors such as lenvatinib, vandetanib, and sunitinib have been reported, the appearance of a drug specifically targeting the RET mutation has displaced other therapies.

### 4.1. RET-Mutated MTC

Both selpercatinib and pralsetinib specifically target RET-mutated MTCs. In the neoadjuvant setting, published results are limited to the former. In 2021, the group at The University of Texas M. D. Anderson Cancer Center [78] published the first case treated with a neoadjuvant intent with successful locoregional surgical salvage of a patient initially considered unresectable. This same group subsequently published a series with four cases treated with selpercatinib that could be surgically rescued, with a good outcome after surgery [79]. A prospective trial to validate these findings is currently ongoing (NCT33169506).

### 4.2. RET-Wildtype MTC

In these cases, the absence of a specific molecular target generally leads to the use of multikinase inhibitors. There is a case report regarding a patient with an advanced thyroid carcinoma, initially diagnosed as an ATC, which, after failure to respond to two lines of chemotherapy with associated radiotherapy, was treated with sunitinib [80]. After 18 months with this treatment, the tumor was finally considered resectable, and a thyroidectomy was performed. The final histology revealed an MTC instead an ATC, and although there were no RET-activating mutations, a Leu769Leu (2307T > G) polymorphism was found. Similarly, a study from Slovenia reports a 50% surgical salvage rate in initially unresectable patients treated with sunitinib [81].

A recent study from Latin American [82] included six cases of MTC; five of them were treated with vandetanib, with a reduction in tumor diameter of 24.5%, but only one patient achieved R0/R1 resection.

Finally, another case report [83] described the case of a patient with a locally advanced tumor treated with lenvatinib. After 4 months of treatment, a reduction in tumor size of 70% was achieved, allowing a complete surgical resection, with no residual disease at 6 months after surgery and a decrease of 99% in serum calcitonin regarding basal levels.

## 5. New Therapeutic Approaches

Several drugs are currently under investigation for the treatment of thyroid cancer, and we will highlight the most notable ones. It would be interesting to assess in the future whether they are effective in cases of advanced thyroid cancer as neoadjuvant therapy.

Chimeric antigen receptor T-cell (CAR-T) therapy has demonstrated efficacy in B-cell malignancies [84]. Evidence in solid tumors is limited, though, in the specific case of thyroid cancer, preclinical studies have shown promising results [85] using TSHR as a target antigen, as well as other antigens such as ICAM-1 [86,87].

Moreover, the use of nanoparticles conjugated to ^131^I has been proposed as photothermal and photodynamic therapy, and this approach has been evaluated in preclinical studies [88].

Finally, galectin-3 inhibitors have also been proposed as a potential treatment in preclinical studies due to their effects on the thyroid cancer cells’ invasive ability [89].

## 6. Conclusions

Locally advanced thyroid carcinoma is a complex and rare situation that should be treated by multidisciplinary teams in specialized centers. The low evidence available warrants the maintenance of prospective registries to clarify the best management of these conditions.

In all three scenarios, the presence of certain mutations will allow for targeted treatment, with a much higher success rate than that offered by multitargeted drugs. Likewise, the emergence in recent years of multiple studies with different therapeutic regimens requires validation by other groups that obtain similar results in order to consider these therapies as part of the usual therapeutic arsenal.
